# Chest pain in the emergency department: risk stratification with Manchester triage system and HEART score

**DOI:** 10.1186/s12872-015-0049-6

**Published:** 2015-06-11

**Authors:** Luís Leite, Rui Baptista, Jorge Leitão, Joana Cochicho, Filipe Breda, Luís Elvas, Isabel Fonseca, Armando Carvalho, José Nascimento Costa

**Affiliations:** Departament of Cardiology, Centro Hospitalar e Universitário de Coimbra, Praceta Prof. Mota Pinto, Coimbra, 3000-075 Portugal; Department of Internal Medicine, Centro Hospitalar e Universitário de Coimbra, Praceta Prof. Mota Pinto, Coimbra, 3000-075 Portugal; Emergency Department, Centro Hospitalar e Universitário de Coimbra, Praceta Prof. Mota Pinto, Coimbra, 3000-075 Portugal

**Keywords:** Chest pain, Emergency department, Manchester triage system, HEART score, Acute coronary syndrome, Angina pectoris

## Abstract

**Background:**

Fast and accurate chest pain risk stratification in the emergency department (ED) is critical. The HEART score predicts the short-term incidence of major adverse cardiac events (MACE) in this population, dividing it in three risk categories. We aimed to describe the population with chest pain, to characterize the subgroup of patients with acute coronary syndrome (ACS) and to assess the prognostic value of Manchester triage system and of HEART score.

**Methods:**

Retrospective observational study including patients admitted to the ED of a tertiary hospital with chest pain as the presenting symptom. The primary outcome was a composite of all-cause mortality, myocardial infarction or unscheduled revascularization at 6 weeks.

**Results:**

We enrolled 233 patients (age 58 ± 19; 55.4 % males). The most common final diagnosis was non-specific chest pain (*n* = 86, 36.9 %), followed by ACS (*n* = 22, 9.4 %). Male gender, smoking and chronic kidney disease were associated with higher risk of ACS. According to Manchester triage system, chest pain patients stratified with red or orange priority had a higher incidence of ACS (16.5 % vs. 3.8 %, *p* = 0.006). The application of HEART score showed that most patients were in low risk category (56.3 %). The six-week incidence of MACE in each category was 2 %, 15.6 % and 76.9 % (*p* < 0.001). HEART score accurately predicted the short-term incidence of MACE in chest pain patients (c-statistic 0.880; 95 % CI, 0.807–0.950, *p* < 0.001).

**Conclusions:**

Chest pain patients have very different levels of severity and the discriminatory power of Manchester triage system should be used in the assessment of this population. The HEART score seems to be an effective tool for risk stratification in the ED.

## Background

Chest pain management is one of the biggest challenges in the emergency department (ED). This symptom accounts for 5 to 20 % of all ED admissions [1], being the second most common reason to present to the ED in the United States of America [2]. Causes of chest pain range from musculoskeletal chest pain to potentially life-threatening emergencies as acute coronary syndrome (ACS), aortic dissection or pulmonary embolism. Therefore, accurate and fast risk stratification is paramount in the acute management of these patients, mainly to identify those patients with immediate risk of complications, as those with an ACS. This group of patients is challenging to discriminate, as there is a variety of clinical manifestations.

To minimize this problem, several risk stratifying tools have been developed in the last years. Some of these tools are applicable to all clinical situations presenting to the ED, as the Manchester triage system. This validated system is performed by specifically trained nurses and prioritize patients according to illness severity and is based on the main complaint and in the presence of certain discriminators, according to a structured questionnaire [3]. A colour is assigned to each level of urgency, specifying a target time to first medical observation: red (immediate); orange (<10 min); yellow (<60 min); green (<120 min) and blue (<240 min).

Other systems, more selective, are devoted to the risk stratification of suspected ACS in the ED. One is the HEART score, designed to be performed by the ED physician, predicting the short-term occurrence of major adverse cardiac events (MACE) - all-cause death, myocardial infarction or revascularization – in the ED population of chest pain patients [4].

We aimed (1) to describe an unselected population of chest pain patients presenting to the ED; (2) to characterize the subgroup of patients diagnosed with an ACS and (3) to determine the value of Manchester triage system and HEART score for risk stratification in acute chest pain.

## Methods

### Study design

We conducted a retrospective cohort study, which included all patients admitted to the ED of a tertiary referral hospital, with non-traumatic chest pain as the chief complaint. This ED receives around 400 to 500 patients daily, having no dedicated chest pain unit. There was no exclusion criterion in the selection of patients.

All clinical, laboratory and imaging data were recorded in a proprietary software specially developed for ED environments (ALERT®, ALERT systems, Vila Nova de Gaia, Portugal). In our ED, all patients are stratified by priority using a risk stratification protocol - the Manchester triage system - performed by specifically trained nurses. From the data introduced in ALERT®, we collected clinical information from the ED patients, analysed the level of priority given, prior medical history, diagnostic tests results and the final diagnosis. According to the diagnosis made by the ED cardiologist, using the current definition [5], patients were divided into two groups: group 1 – established ACS diagnosis; group 2 – no ACS. Nevertheless, every patient admitted to in-hospital care was reviewed by the authors to confirm the diagnosis. The research protocol was approved by the local ethics committee (*Comissão de Ética para a Saúde do Centro Hospitalar e Universitário de Coimbra*).

### ACS risk-stratifying systems

The HEART score was retrospectively applied to the population according to the information available in the ALERT® system and in the electronic health records of the hospital. The acronym HEART is an abbreviation of the five parameters evaluated [6]:

- History (highly suspicious – 2 points; moderately suspicious – 1 point; slightly suspicious – 0 points)
- ECG (significant ST-depression – 2 points; non-specific repolarization disturbance – 1 point; normal – 0 points)
- Age (≥65 years – 2 points; 45–65 years – 1 point; ≤ 45 years – 0 points)
- Risk factors for coronary heart disease (≥3 risk factors – 2 points; 1 or 2 risk factors – 1 point; no risk factors – 0 points)
- Troponin (≥3 times the threshold for positivity – 2 points; 1 to 3 times the threshold for positivity – 1 point; normal limit – 0 points)

Two independent authors, blinded to the final diagnosis, performed the classification of the degree of suspicion in the ‘history’ parameter. In the cases with two different scores, the mean value was considered. The ECG’s were interpreted by the attending physician in the ED, specialist in internal medicine or in cardiology.

According to the total score received, patients are divided into low (0–3), intermediate (4–6) or high (7–10) risk of a MACE, within 6 weeks after presentation at the ED. Lower scores lead to a recommendation of early discharge, intermediate scores suggest clinical observation or performance of non-invasive investigations and high scores admission for more invasive strategies [4]. In the analysis of HEART score data, we only used patients with available records to complete all the five parameters of the score, excluding patients without ECG or troponin measurement. This has led to a dropout of 59 patients (25.3 %).

A 6-week follow-up of all patients was conducted through the electronic health records information of the hospital and through direct phone calls to the patients involved. The follow-up focused on the composite endpoint of MACE, comprising all-cause death, myocardial infarction or unscheduled revascularization. No patient was lost to follow-up.

### Statistical analysis

We aimed to find a difference in 6-week MACE between low, intermediate and high risk categories of HEART score. Based on the proportions of the multicentre study of Backus et al. [4] and assuming an alpha of 0.05, we would need a sample of 168 patients to detect differences with 80 % power. Since we anticipate that 20–25 % of patients could not have records to complete all the parameters of the score, we aimed for a sample size of 220 patients. The period of time analysed included 1 week from winter (23rd to 29th January 2012) and 1 week from summer (23rd to 29th July 2012), randomly selected, in order to balance the list of final diagnosis. Statistical analysis was carried out using IBM SPSS Statistics for Windows software version 20.0® (Armonk, New York). Normality of continuous variables was tested by histogram observation and Kolmogorov-Smirnov test. Continuous variables are presented by mean ± standard deviation and categorical variables as percentage. We used Student’s *T* test and Mann–Whitney test for comparison of means for continuous variables, Chi-square and Fisher’s exact test for comparison of categorical variables. Multiple logistic regression adjusted for confounding factors was performed considering any variable with *p* < 0.25 in univariate analysis. A received operator characteristics (ROC) curve was used to determine the discriminatory power of HEART score. Results with *p* < 0.05 were considered as statistically significant.

## Results

### Characterization of the chest pain population in the ED

We evaluated 233 patients presenting with non-traumatic chest pain, which accounted for 4.2 % of all ED admissions. The demographic characteristics and the priority stratification according to Manchester triage system are shown on Table 1. Age ranged from 19 to 98 years and women were significantly older than men (60.7 ± 20 vs. 55.4 ± 13, *p* = 0.012).

**Table 1:** Demographic characteristics and Manchester triage system

Variable	
Age, mean ± SD	57.7 ± 19
Male gender, n (%)	129 (55.4)
Manchester triage system, n (%)	
- Red	1 (0.4)
- Orange	102 (43.8)
- Yellow	99 (42.5)
- Green	31 (13.3)
Past medical history, n (%)	
- Hypertension	125 (53.6)
- Dyslipidemia	90 (38.6)
- Type 2 diabetes	33 (14.2)
- Active smoker	31 (13.3)
- Coronary artery disease	36 (15.5)
- Atrial fibrillation	42 (18)
- Heart failure	38 (16.3)
- Chronic pulmonary obstructive disease	16 (6.9)
- Chronic kidney disease	11 (4.7)
- Others	31 (13.3)

*SD* standard deviation

The most frequently requested diagnostic tests were chest X-ray (85.4 %), ECG (81.5 %) and cardiac biomarkers (74.2 %). The median time from patient ED arrival to ECG acquisition was 20 (IQR 12–41) minutes. According to Manchester triage system, patients stratified with a red or orange priority performed the ECG significantly faster than those with yellow or green priority (median 15 vs. 28 min, *p* < 0.001)

**Figure 1:**
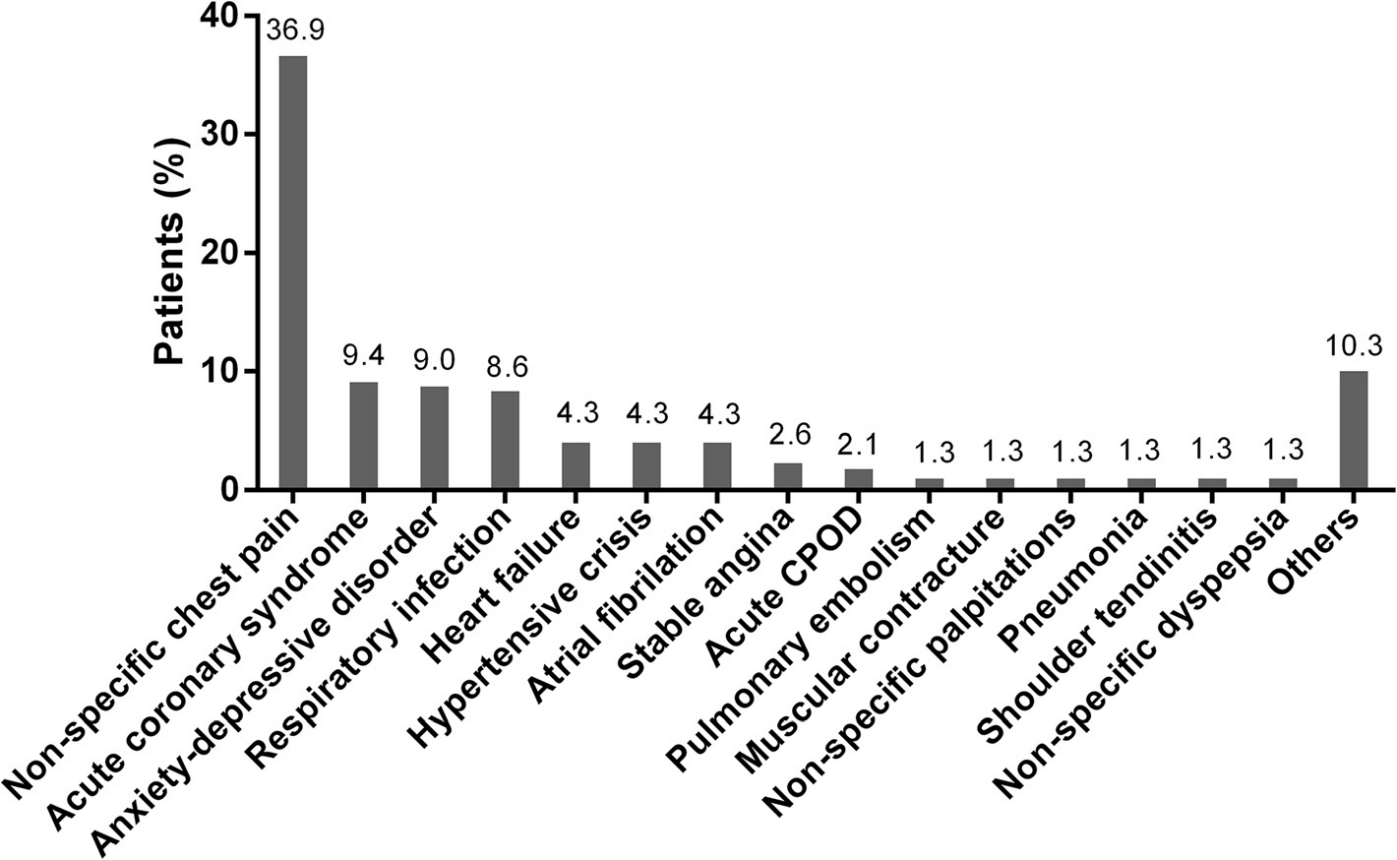
Final diagnosis of patients. COPD, chronic obstructive pulmonary disease

As presented in Fig. 1, after clinical observation and interpretation of diagnostic tests, the most common diagnosis made by the attending physician was non-specific chest pain (*n* = 86, 36.9 %), followed by ACS (*n* = 22, 9.4 %), anxiety-depressive disorder (*n* = 21, 9.0 %) and respiratory infection (*n* = 20, 8.6 %). The group of the less frequent diagnosis (10.3 %) included: cardiovascular diseases, as aortic dissection or acute pericarditis; pulmonary diseases, as acute asthma; and gastrointestinal diseases, as gastro-oesophageal reflux disease or biliary colic.

Stratifying the final diagnosis by season of the year, we found a higher proportion of patients being diagnosed with respiratory infections in the winter than in summer (15.9 % vs. 1.7 %, *p* <0.001), whereas the proportion of the other final diagnosis, like non-specific chest pain or ACS, were similar in both seasons.

The great majority of patients presenting in the ED with chest pain were discharged home (*n* = 189, 81.1 %), while the remaining were admitted to hospital (*n* = 44, 18.9 %). The incidence of the composite endpoint of MACE was 9.4 %.

**Figure 2:**
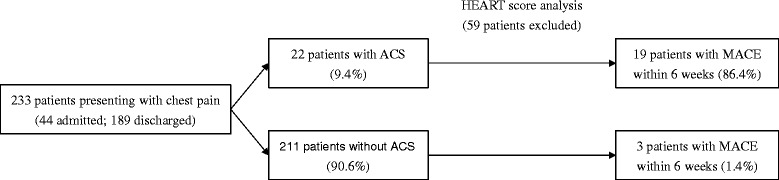
Flow diagram of patients presenting with chest pain. ED, emergency department; ACS, acute coronary syndrome; MACE, major adverse cardiovascular events

The patient flow diagram is summarized in Fig. 2.

### Subgroup of acute coronary syndrome patients

Among the group of patients with a final diagnosis of ACS (group 1, *n* = 22), the most common ACS subtype was unstable angina (10 patients - 46 %), followed by non-ST-elevation myocardial infarction (NSTEMI) (7 patients - 32 %) and ST-elevation myocardial infarction (STEMI) (5 patients - 23 %). Group 1 patients were older and had a higher proportion of males, compared with the group of patients without ACS. Moreover, they had a significantly higher prevalence of hypertension, type 2 diabetes, dyslipidaemia, smoking habits, previously known coronary artery disease and chronic kidney disease (Table 2).

**Table 2:** Comparative of population characteristics ACS versus no ACS

	Group 1 – ACS (*n* = 22)	Group 2 – No ACS (*n* = 211)	*p*
Age, mean ± SD	65.6 ± 15	56.9 ± 19	0.043
Male gender, n (%)	18 (81.8)	111 (52.6)	0.012
Hypertension, n (%)	18 (81.8)	107 (50.7)	0.006
Dyslipidemia, n (%)	17 (73.9)	73 (34.6)	0.001
Type 2 diabetes, n (%)	8 (36.4)	25 (11.8)	0.005
Smoking, n (%)	8 (36.4)	23 (10.9)	0.003
Coronary artery disease, n (%)	9 (40.9)	27 (12.7)	0.002
Atrial fibrillation, n (%)	3 (13.6)	39 (18.5)	0.773
Heart failure, n (%)	3 (13.6)	35 (16.6)	0.957
Chronic pulmonary obstructive disease, n (%)	1 (4.5)	16 (7.6)	0.376
Chronic kidney disease, n (%)	5 (22.7)	6 (2.8)	0.002

*ACS* acute coronary syndrome, *SD* standard deviation

The great majority of patients diagnosed with an ACS were given high priorities by the Manchester triage system, with only 1 patient given the colour green (4.5 %). Most patients were classified as orange (*n* = 16, 72.8 %), followed by yellow (*n* = 4, 18.2 %) and red (*n* = 1, 4.5 %). The five STEMI patients were triaged with orange (*n* = 4, 80 %) or red (*n* = 1, 20 %) priority, while the seven NSTEMI patients were triaged as orange (*n* = 6, 85.7 %) or green (*n* = 1, 14.3 %).

There was a correlation between Manchester’s priority category in chest pain patients and the final diagnosis of ACS: 3.2 % in the green category, 4.0 % in the yellow category, 15.7 % in the orange category and 100.0 % in the red category. Patients stratified red or orange had a significantly higher incidence of ACS compared with those with yellow or green levels of priority (16.5 % vs. 3.8 %, *p* = 0.006).

Using a multivariate analysis model controlling for age, gender, hypertension, dyslipidaemia, type 2 diabetes, smoking, coronary artery disease and chronic kidney disease, male gender (odds-ratio (OR) 4.48; 95 % confidence interval (CI), 1.12–17.40; *p* = 0.030), smoking (OR 4.22; 95 % CI, 1.21–14.74; *p* = 0.024) and chronic kidney disease (OR 8.21; 95 % CI, 1.76–38.16; *p* = 0.007) emerged as independent predictors of ACS in this population.

### The use of HEART score in risk stratification

The determination of the HEART score in the population with complete datasets (*n* = 174) showed that most chest pain patients were low risk (*n* = 98, 56.3 %), followed by intermediate (*n* = 64, 36.7 %) and high risk (*n* = 12, 6.9 %).

**Figure 3:**
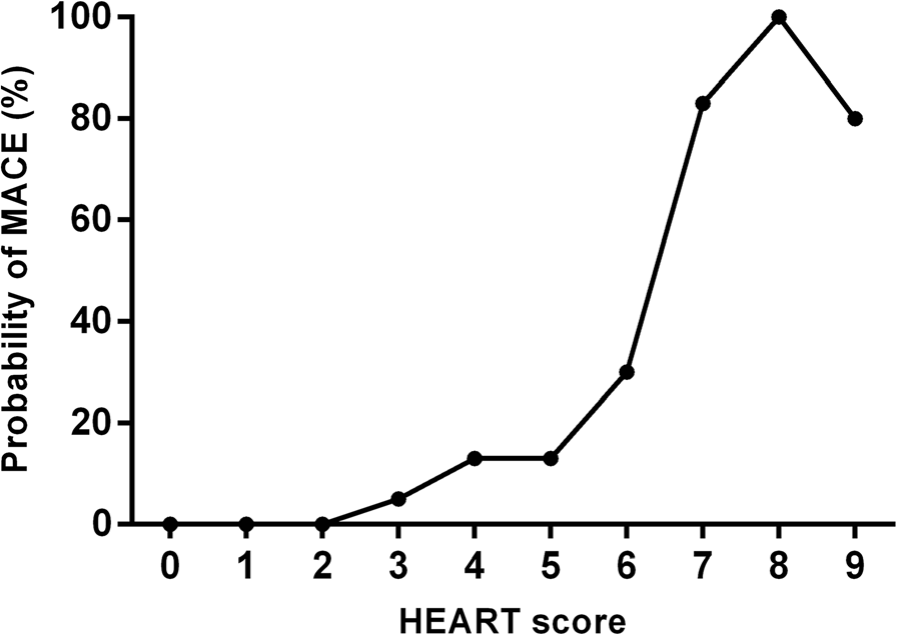
Incidence of 6-week MACE in each HEART score. MACE, major adverse cardiovascular events

Figure 3 illustrates the relationship between the HEART score and the incidence of the composite endpoint, with higher scores associated with higher incidence of 6-week MACE.

**Figure 4:**
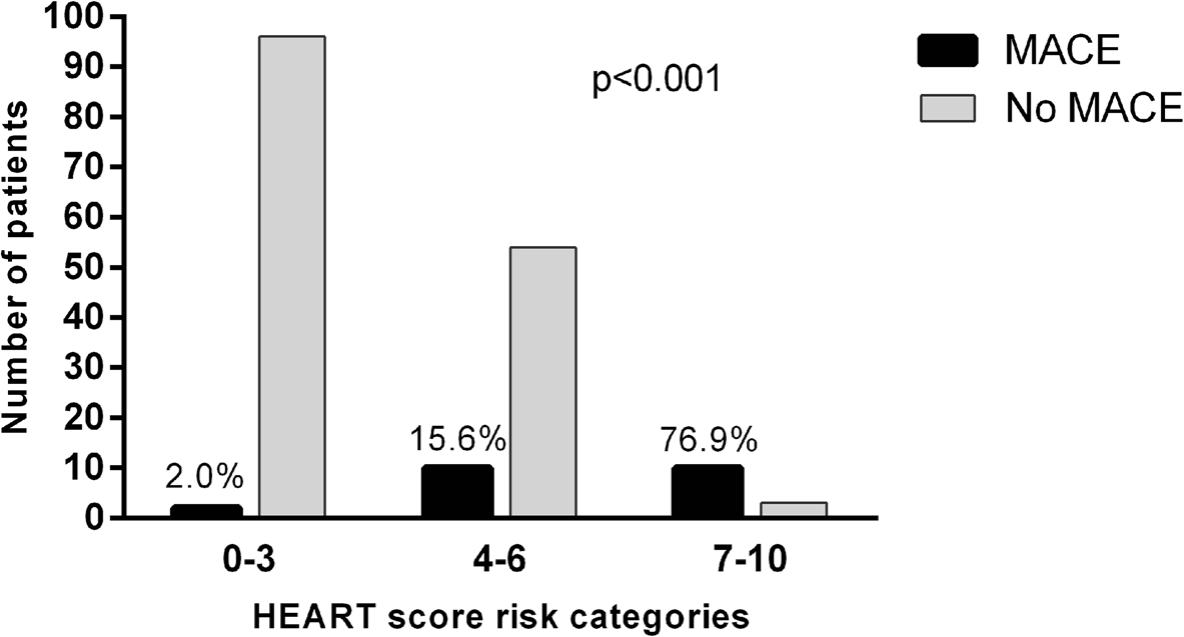
Incidence 6-week MACE in each HEART score risk category. MACE, major adverse cardiovascular events

When the three externally validated categories of the HEART score were used (low risk 0–3; intermediate risk 4–6; high risk 7–10), a good discrimination of the incidence of 6-week MACE was obtained (2 %, 15.6 % and 76.9 %, *p* < 0.001), as showed in Fig. 4.

The HEART score had a good discriminatory power (c-statistic 0.880; 95 % CI, 0.807 – 0.950, *p* < 0.001) to predict the probability of the composite endpoint. The sensitivity, specificity, positive and negative predictive values for the established cut-off scores of 4 and 7 are shown in Table 3.

**Table 3:** Sensitivity, specificity, positive and negative predictive values for six-week incidence of MACE using HEART score

	Cut-off = 4^a^	Cut-off = 7^b^
Sensitivity, % [95 % CI]	90.9 [70.8; 98.9]	45.5 [24.4; 67.79]
Specificity, % [95 % CI]	63.2 [55.0; 70.8]	98.7 [95.3; 99.8]
Positive predictive value, % [95 % CI]	26.3 [16.9; 37.7]	83.3 [51.59; 97.9]
Negative predictive value, % [95 % CI]	97.9 [92.8; 99.8]	92.6 [87.4; 96.1]

^a^98 patients with score <4 and 76 with score ≥4

^b^162 patients with score <7 and 12 with score ≥7

## Discussion

In this study we described a population of patients presenting to an ED with chest pain as their main complaint. We demonstrated that less than 10 % have an ACS as the aetiology basis of this symptom and that the Manchester triage system correctly prioritized the patients with most severe causes of pain. Finally, we retrospectively tested the HEART score in our population, demonstrating that it has a good discriminatory power to identify the risk of MACE in a short term period.

Our sample presented a male predominance, a wide range of age and a mean age similar to previous studies [7–9]. The analysis of the Manchester triage system in this population showed that more than a half of the patients were stratified with a green or a yellow level of severity, in accordance with the high incidence of potentially benign causes of chest pain. The most frequent comorbidities were similar to other studies [7, 9]; however we found a higher prevalence of hypertension and lower proportion of active smokers.

Considering the diagnostic tests, and in comparison with the results of the Spanish study of Martínez-Sellés et al. [7], our ED physicians requested more chest X-rays and cardiac biomarkers in this sample. The ECG is a first-line diagnostic tool in chest pain assessment and the time from ED arrival to ECG acquisition in this cohort was greater than the 10 min recommended in the current European Society of Cardiology guidelines [5]. Therefore, an effort must made to shorten this time, in order to identify life-threatening emergencies, namely to reduce time to revascularization therapy in STEMI.

The final diagnostic list of this population was extensive, with very different levels of severity, as in the literature [10]. Non-specific chest pain (mostly musculoskeletal pain) was the most frequent diagnosis, but with a proportion slightly lower than that reported in other studies, were it ranges from 43 to 59 % [7, 11]. This diagnosis is thus probably the most frequent one in an unselected population. ACS represented the second most common final diagnosis of chest pain in our population, albeit with a lower percentage than that reported in the literature (15.7 %) [7]. Stable angina had a relatively low proportion (2.6 %) on the list of the final diagnosis. Although this percentage is only slightly lower than in other studies [7], this clinical presentation of coronary artery disease may be underdiagnosed as non-specific chest pain.

Our study reports some traditional cardiovascular risk factors [12] that can increase significantly the odds of chest pain being of unstable coronary artery disease origin, as male gender, smoking and chronic kidney disease.

Considering the Manchester triage system, most of ACS patients were stratified with an orange or red level of priority, but this percentage was slightly lower than in previous studies [13, 14]. Although the proportion of patients stratified as yellow or green (*n* = 5, 22.7 %) could lead to an important delay in the management of ACS, namely in the time from arrival to ECG acquisition, none of these cases were STEMI, a reassuring finding that highlights the clinical value of the triage system. Taking in consideration all chest pain patients in the ED, the proportion of ACS is significantly higher in orange or red priority patients, in comparison to green or yellow, showing that the Manchester triage system has a reasonable discriminatory power. Our data are in line with another Portuguese cohort, where the sensitivity of this system in assigning red or orange priority to patients with ACS was 87.3 % [13].

In search for the optimal risk stratifying system for chest pain patients, we analysed the HEART score. This score was developed as an attempt to create an easy-to-use ACS risk quantification, incorporating well known markers of increased risk available on the ED, allowing a more firmly based decision, mainly in cases of atypical presentation or absence of ECG abnormalities [6]. The application of the HEART score to our population showed that the majority of patients are low risk, as it was reported in the first cohort where this score was applied [6]. The relationship between the HEART category and adverse outcomes, defined as the occurrence of MACE within 6 weeks, showed a curve with three different patterns, corresponding to the three risk categories defined by the literature [15]. The risk stratification using the three categories (0–3; 4–6; 7–10) identified MACE with similar proportions than in the multicentre study of Backus et al. [4], but with greater risk of MACE in the high risk category.

Our study confirmed the appropriate discriminatory power of the three risk categories of HEART score to predict short-term occurrence of MACE. A score <4, corresponding to low risk category, had a very high negative predictive value, identifying a small risk population. Moreover, the high risk category (score ≥7) showed a reasonable positive predictive value, allowing the identification of a high risk population, even in patients with more atypical presentations. This risk score may help in making accurate management choices in a setting that is frequently denoted by uncertainty, by being a strong predictor of event free survival and of potentially life threatening cardiac events.

The limitations of this study are mainly those inherent to single-centre retrospective studies. The prevalence of obesity, an important cardiovascular risk factor, was not analysed because of lack of data about body mass index. In the analysis of HEART score, we only used patients with all the five parameters available to complete the score, which could lead to a selection bias. However, when comparing the characteristics of the patients with incomplete datasets with patients with complete datasets, no differences were found in the available variables. We are unaware of studies where the HEART score has been applied prospectively in real time, but such studies would be useful to confirm the prognostic value of the score. Furthermore, during the period of time analysed, the laboratory was using conventional troponin I assays, so we could not evaluate the potential impact of high-sensitivity troponin in chest pain management.

## Conclusions

Chest pain is a common complaint in the ED and has an extensive differential diagnosis list with very different levels of severity. Age, gender, traditional cardiovascular risk factors and the priority attributed by the Manchester triage system should be considered in the assessment of patients. Moreover, the HEART score seems to be a useful tool for risk stratification and decision making in acute chest pain.

## Abbreviations

ACS: Acute coronary syndrome
CI: Confidence interval
ED: Emergency department
MACE: Major adverse cardiac events
NSTEMI: Non-ST-elevation myocardial infarction
OR: Odds-ratio
SD: Standard deviation
STEMI: ST-elevation myocardial infarction
